# Developing Mobile Health Applications for Inflammatory Bowel Disease: A Systematic Review of Features and Technologies

**DOI:** 10.34172/mejdd.2024.394

**Published:** 2024-10-30

**Authors:** Parvin Akbarian, Farkhondeh Asadi, Azam Sabahi

**Affiliations:** ^1^Department of Health Information Technology and Management, School of Allied Medical Sciences, Shahid Beheshti University of Medical Sciences, Tehran, Iran; ^2^Department of Health Information Technology, Ferdows Faculty of Medical Sciences, Birjand University of Medical Sciences, Birjand, Iran

**Keywords:** Mobile application, Portable software apps, Inflammatory bowel diseases

## Abstract

**Background:**

Patients with inflammatory bowel disease (IBD) require lifelong treatment, which significantly impacts their quality of life. Self-management of this disease is an effective factor in managing chronic conditions and improving patients’ quality of life. The use of mobile applications is a novel approach to providing self-management models and healthcare services for patients with IBD. The present systematic review aimed to identify the features and technologies used in the development of IBD disease management applications.

**Methods:**

This systematic review was conducted according to PRISMA guidelines in PubMed, Scopus, and Web of Sciences databases up to August 8, 2023, which included initial searches, screening studies, assessing eligibility and risk of bias, and study selection. The data extraction form was based on the study objectives, including bibliographic information from articles, such as the first author’s name, year of publication, country of origin, and details related to mobile health applications, such as the name of the application, features and technologies used, advantages and disadvantages, main outcomes, and other results. The content of the research was analyzed according to the research objectives.

**Results:**

In the initial review of four databases, a total of 160 articles were retrieved and subsequently entered into EndNote. After removing duplicates and irrelevant studies based on title, abstract, and full-text assessments, 12 articles were finally selected. The studies were conducted between the years 2015 and 2024. 100% of the applications designed for patients with IBD were aimed at treatment, 83% were for self-management of the disease, and 33% of the applications were intended for disease diagnosis. The features of IBD management applications were categorized into four groups: education, monitoring, counseling, and diagnosis and treatment.

**Conclusion:**

Various mobile applications have been developed for the management of IBD, each differing in features and technologies used. While current IBD applications have limited capabilities in diagnosing disease severity, they still hold significant potential in empowering patients through education, counseling, and monitoring. The integration of artificial intelligence and decision support systems may enhance the effectiveness and reliability of these applications.

## Introduction

 Inflammatory bowel disease (IBD) is a comprehensive term for a set of chronic diseases, primarily including Crohn’s disease and ulcerative colitis.^1^ This condition is characterized by changes in the composition and function of the gut microbiome and systemic biochemical abnormalities, manifesting not only in the intestines and gastrointestinal tract but also in many patients in extraintestinal organs, often affecting the joints, skin, and eyes, which can occur before or after the diagnosis.^2-4^ Most patients require lifelong treatment, which significantly impacts their quality of life and survival while also creating an economic burden due to direct and indirect healthcare costs.^5,6^ IBD has long been regarded as a public health threat in Western countries, but the increasing incidence of IBD in developing countries has turned it into a global issue in recent decades.^7^ IBD can significantly affect individuals’ lives, as well as their physical, psychological, emotional, and social well-being.^8^ With projected prevalence rates in some areas reaching 1% over the next decade and the ongoing rise in IBD prevalence alongside an aging population worldwide, the use of healthcare resources by patients with IBD is inevitably increasing.^9^

 The primary concerns of patients with IBD include reduced quality of life, medication side effects, unpredictability, psychological and physical complications, social isolation, the need for surgery, and the risk of cancer.^10,11^ While large academic IBD centers may provide multidisciplinary support from nutritionists, psychologists, therapists, and pharmacists, many patients do not have access to these services and report a need for dietary and lifestyle interventions.^12^ Given the complexity and lifelong nature of this disease, patient ownership and engagement in their disease management are crucial for improving quality of life, preventing disease complications, and enhancing disease self-management.^13^ Today, patients are increasingly seeking to participate in treatment decision-making and disease management based on their preferences, which positively impacts reported quality of life, long-term adherence, and resource utilization.^5^ Self-management strategies are effective due to the chronic and unpredictable nature of IBD, enabling patients to take control of their lives and health.^14^

 Electronic health (e-Health) encompasses the use of technologies that facilitate communication between patients and healthcare providers, providing a solution for more effective management of IBD care beyond the clinical setting, both in terms of patient outcomes and cost reduction.^15,16^ A study by Wang and colleagues showed that patients who use smartphone applications to monitor their medical conditions feel more secure about their status, engage more in their health, and believe they are well cared for outside the clinical environment.^17^ However, there is an increasing need to initiate a digital transition in the medical management of IBD to achieve multifaceted clinical benefits, from enhanced treatment adherence to precise monitoring, with feasibility in terms of economic sustainability and reduced healthcare costs.^17^ Many studies have examined the impact of mobile health on healthcare utilization for patients with IBD, demonstrating that mobile health management leads to reduced outpatient visits and hospitalizations, less use of injectable medications, fewer endoscopic procedures, and decreased healthcare-related costs.^18^

 Previous studies have primarily focused on applications’ content and the needs of their audience. They revealed that IBD management applications lack professional medical intervention, adherence to guidelines, and clinical credibility, which poses challenges for physicians. They recommended that the development of future apps prioritizes behavioral change models and understanding the needs of both patients and physicians to effectively meet the individual needs of patients with IBD.^19-21^

 Preliminary reviews by the authors indicated that no systematic review focusing on the features and technologies has been conducted to date. Therefore, given mobile health applications’ capabilities in enhancing IBD management and providing better patient outcomes, identifying the necessary features and technologies in mobile health applications can assist developers in better design. This study was conducted to examine mobile applications developed for managing IBD, focusing on identifying the features and technologies used in these applications.

## Materials and Methods

###  Study Design and Search Strategy

 This systematic review was conducted according to the Preferred Reporting Items for Systematic Reviews and Meta-Analyses (PRISMA) until August 8, 2023, which included the initial search, screening of studies, assessing the eligibility and risk of bias, and study selection.^22^ The searches were done using a combination of keywords, which are indicated in Table 1.

**Table 1:** Search strategies for different databases

**Database**	**Keywords**
PubMed	((“Mobile Application*” [Title/Abstract] OR “Mobile Application*” [Mesh terms] OR “Portable Electronic App*” [Title/Abstract] OR Portable Electronic [Title/Abstract] OR “Electronic Apps” [Title/Abstract] OR “Portable Software Apps” [Title/Abstract] OR “Portable Software” [Title/Abstract]) AND (“Inflammatory Bowel Diseases” [Mesh term] OR “Inflammatory Bowel Diseases” [Title/Abstract] OR “Inflammatory Bowel Disease” [Title/Abstract] OR “Bowel Diseases” [Title/Abstract]))
Scopus	((TITLE-ABS-KEY (“Mobile Application*”) OR TITLE-ABS-KEY (“Portable Electronic App*”) OR TITLE-ABS-KEY (“Portable Electronic”) OR TITLE-ABS-KEY (“Electronic Apps”) OR TITLE-ABS-KEY (“Portable Software Apps”) OR TITLE-ABS-KEY (“Portable Software”)) AND (TITLE-ABS-KEY (“Inflammatory Bowel Diseases”) OR TITLE-ABS-KEY (“Inflammatory Bowel Disease”) OR TITLE-ABS-KEY (“Bowel Diseases”)))
Web of Science	((TS= (“Mobile Application*”) OR TS= (“Portable Electronic App*”) OR TS= (“Portable Electronic”) OR TS= (“Electronic Apps”) OR TS= (“Portable Software Apps”) OR TS= (“Portable Software”)) AND (TS= (“Inflammatory Bowel Diseases”) OR TS= (“Inflammatory Bowel Disease”) OR TS= (“Bowel Diseases”)))

###  Data Sources

 To find the studies, scientific databases, including PubMed, Scopus, and Web of Sciences, were searched according to the search strategy, and the found articles were imported into the EndNote reference manager software (EndNote X20, Clarivate Analytics, Philadelphia). Additionally, to retrieve other related studies, a hand search was made in the list of references of the included articles and the Google Scholar search engine with different combinations of keywords.

###  Study Selection Process

 The selection criteria comprised original articles in English that introduced or reviewed health applications for IBD, specifically examining the features and technologies used in these applications. This study excluded brief reports, letters to the editor, conference abstracts, review articles, and articles that were not available in full text or written in a language other than English.

###  Data Extraction

 The process of data extraction involved utilizing a researcher-made data extraction form in Excel (Microsoft, 2019) based on the objectives of the study after selecting the studies. This form consisted of bibliographic information of articles, such as the first author’s name, publication year, country of origin, and specifics regarding mobile health applications, such as the name of the application, features, and technologies used in these applications, the advantages and disadvantages, the main outcome, and other outcomes. The content of the study was then analyzed, considering the study’s aim.

###  Quality and Risk of Bias Assessment

 The quality assessment of studies was done using the Newcastle-Ottawa Scale (NOS), a widely recognized tool for assessing the quality of observational studies.^23^ Some modifications were made to NOS to accommodate the assessment of the quality of health information systems in studies. All articles had the necessary quality according to the checklist.

###  Data Synthesis and Analysis

 The descriptive analysis, which included frequency and percentage parameters, was calculated based on the study’s variables. The results were then presented in the form of graphs and tables.

## Results

###  Study Selection

**Figure 1:**
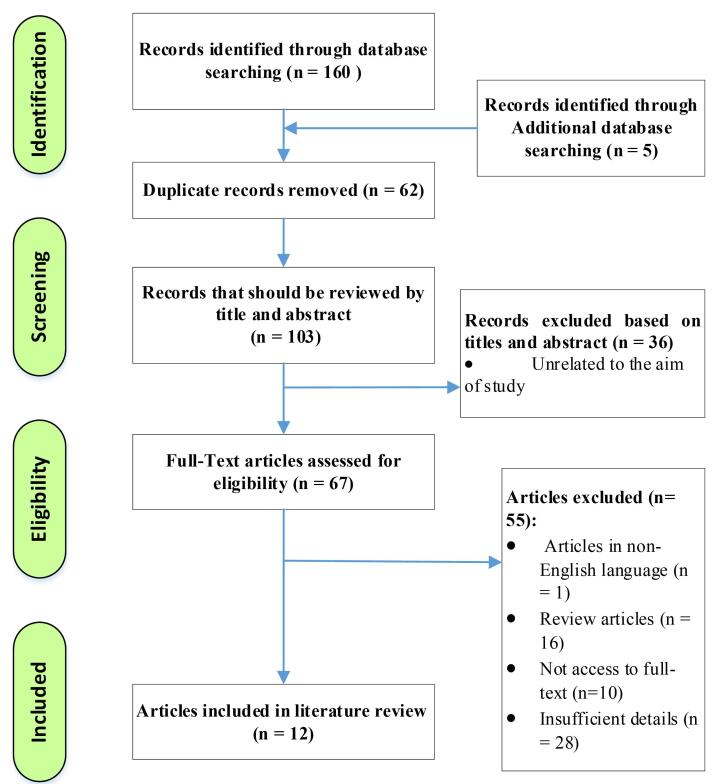
PRISMA flow diagram indicating results of identification and screening process

 In the initial review of four databases, 160 articles were retrieved and then imported into EndNote software. After removing duplicates and irrelevant articles based on title, abstract, and full-text evaluation, 12 articles were ultimately included in this systematic review. The process of screening and selecting the included and excluded articles is presented in Figure 1.

###  Characteristics of the Included Studies

 The main characteristics of the 12 studies are shown in Table 2. The time frame for the studies was between 2015 and 2024. Most studies were conducted in the USA,^16,24,25^ while others were conducted in Romania,^26^ Japan,^27^ Spain,^28^ Belgium,^5^ the Netherlands,^6^ Poland,^29^ Canada,^30^ India,^31^ and the UK.^32^

**Table 2:** Main characteristics of the studies

**First author (reference)**	**The year of the study**	**country**	**Type of IBD**	**Application name**	**Application aim**	**Main results **
Oancea^26^	2022	Romania	Colitis ulcerative & Crohn's disease	IBD Monitor	Maintain communication between patients and doctors	The possibility of obtaining relevant information about the epidemiology of IBD, patient characteristics and awareness of the problems that patients face in complying with prescribed treatments
Song^27^	2023	Japan	Colitis ulcerative & Crohn's disease	Collection of patient-generated health data with a mobile application and transfer to hospital information system (HIS) via QR codes	Encouraging patients to continuously record their daily conditions on a mobile device and the possibility of transferring data to HIS	Using a mobile application to collect patient-generated health data and transfer it to the HIS is an effective and efficient way to improve data accuracy and accessibility in healthcare institutions.
Del Hoyo^28^	2020	Spain	IBD	TECCU App (Telemonitoring of Crohn's Disease and Ulcerative Colitis)	Improving app usability and effectiveness in managing IBD symptoms and enhancing patient care	The app provided a quick and easy platform for patients and providers to use. The design of the platform should favor shared decision-making with healthcare providers through an open and secure communication process.
Coenen^5^	2020	Belgium	IBD	Mynexuzhealth IBD	Demonstrate the effectiveness and accuracy of remote monitoring tools for monitoring disease activity	This application has been successful in active remote continuous monitoring and timely disease diagnosis.
de Jong^6^	2017	Netherlands	IBD	MyIBDcoach	Testing the feasibility of this system	The application enabled the creation of integrated care for all patients with IBD regardless of disease severity or drug use, facilitated communication with healthcare providers, and improved knowledge about IBD using e-learning modules.
Stawiski^29^	2015	Poland	Chronic gastrointestinal disorders, such as chronic pancreatitis, IBD, or irritable bowel syndrome	Pancre App	Development, establishment, and evaluation of an interactive application	This app provided an innovative approach to individualizing nutrition guidelines and utilized collected data to analyze retrospective disease information and create flexible, personalized nutrition models.
Zand^16^	2021	USA	IBD	UCLA eIBD	Examining patients' satisfaction with the application for care management and receiving feedback from them in order to improve the mobile application	This app improved patient experience and satisfaction and provided useful recommendations for future e-health solutions.
Erlich^30^	2023	Canada	IBD	MyHealthyGut	Meeting the needs of patients with IBD and healthcare practitioners in their care	This app is of value and use or recommended for IBD patients and healthcare providers specializing in this field.
Gupta^31^	2022	India	IBD	IBD NutriCare	Providing a personalized patient profile with a database containing demographic information, medications, daily diet, clinical symptoms and disease scores	A potential tool for telenutrition to improve patient care Acceptable validation of this app against the traditional recall method of dietary assessment of the database provided by the app can be used to fill existing gaps in our knowledge of dietary interventions in IBD.
Fawson^32^	2022	UK	IBD	IBD-BOOST	Addressing patients' concerns by creating an online self-management intervention for fatigue, pain, and urgency/incontinence, as well as meeting the needs of patients	The importance of addressing the specific needs and desires of patients with IBD in the development of online self-management apps for symptoms such as fatigue, pain, and urgency.
Roberts^24^	2024	USA	IBD	SMART IBD	Identification of barriers to self-management in adolescents with IBD	App-based interventions in adolescence will ideally lead to better access and access to self-management interventions as well as improved health behaviors and outcomes in adulthood.
Atreja^25^	2015	USA	IBD	HealthPROMISE	Improving the quality of care and the quality of life of patients with IBD through the implementation of this platform	This platform can be an effective tool in the management and quality of care and quality of life of patients with IBD, increasing the overall well-being of patients with this chronic disease and ultimately empowering patients and providers.

 The results of the present study showed that 100% of the applications designed for patients with IBD were aimed at treatment, 83% were for self-management,^6,16,24-26,28-32^ and 33% of the applications were designed for disease diagnosis.^5,6,16,28^

###  The IBD Management Applications’ Features

 The features of IBD management applications are categorized into four groups: education, monitoring, consultation, and diagnosis (Table 3).

**Table 3:** Features of IBD management applications

**Main groups**	**Subgroups (reference)**
Education	Educational programs include healthy lifestyle habits, including nutrition, exercise, relaxation, and mental health^16^ Nutrition education and instructions for common IBD foods^30^ General dietary recommendations for IBD based on disease status, such as flare or remission^31^ Teachings about ways to deal with fatigue, pain, and urgency^32^ Teaching information about IBD disease^26^
Supervision	Direct monitoring of patient treatment management through connection to the IBD registry^26^ Connecting to HIS and physicians' use of health data effectively to monitor patient-centered medical care^27^ Sending data to electronic medical record (EMR) and generating alerts: review of clinical deviations, flare-ups, and follow-up of alerts by nurses^5,24,25^ Monitoring the exacerbation of the disease, tracking the activity of the disease, and communication between the patient and the doctor^6,31^ Monitor disease activity, schedule appointments, and send messages to providers^16^ Supervision of nutritionists directly on the amount of dietary intake^26^ Enthusiastic monitoring and support of symptom and diet tracking functions and suggestions for finding other sources of advice^32^ Visual reports integrated with electronic health records (EHRs)^25^
Counseling and treatment	Collecting health data generated by the patient to understand the patient's daily condition for efficient treatment^27^ Sending pictures using the phone and automatic system messages according to the needs of patients^28^ Personal response to patients by a nurse or doctor within 24 to 48 hours^28^ Predicting the number of meals with special value for each patient^29^ Sending messages to service providers and scheduling appointments^16^ Patient symptom tracking and provider feedback^32^ ECBT (electronic cognitive behavioral therapy)^16^ Dietary recommendations based on disease conditions such as flare or recovery^31^ Providing evidence-based care^24^ Decision support to create alerts and dashboard reports^5,25,30^
Diagnosis	Matching Eliza laboratory values ​​to combine PRO (Patient Reported Outcome) values ​​with calprotectin home test values^28^ Questionnaire for Crohn's and Colitis patients and investigation of clinically related deviations in data for patients^5^ disease activity tracking^6^

###  The IBD Management Applications’ Limitations

Table 4 demonstrates the limitations of IBD management applications.

**Table 4:** Limitations of IBD disease management applications

**Limitation (Frequency, Percentage) (Reference)**
Difficult implementation on all types of smartphones (n=1, 8.3%)^26^
Inconsistency of user experience due to different screen shapes and different operating systems (n=1, 8.3%)^26^
Security risks when printing due to lack of encryption of data in QR (Quick Response code) codes (n=1, 8.3%)^27^
Dependence of data size in QR codes (Quick Response code) on mobile phone specifications (n=1, 8.3%)^27^
Implementation only in the environment of Microsoft Windows and Android phones (n=1,8.3%) ^29^
Inability to use the app without basic computer skills (n=1, 8.3%)^29^
Internet access (n=1, 8.3%) ^24^
Access to the application only on the iOS platform (n=1, 8.3%)^30^

**Figure 2:**
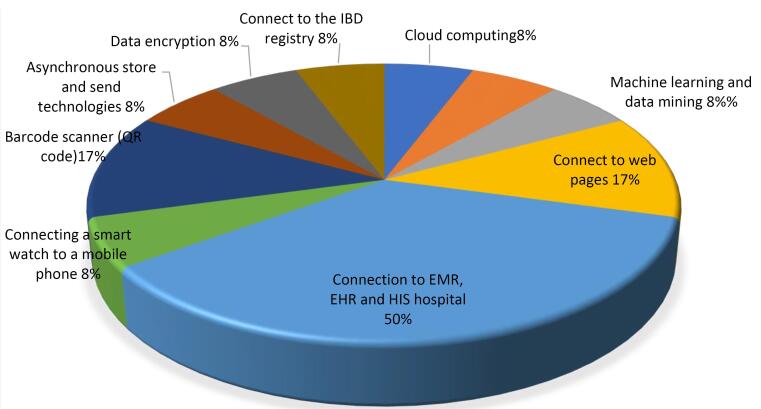
Technologies used in IBD management applications

 The reviewed applications in the studied articles used different technologies, the details of which are given in Figure 2.

## Discussion

 This systematic review examined the features and technologies used to develop IBD management applications. These applications provide comprehensive monitoring tools that allow patients to track their symptoms, medication adherence, and any side effects. This real-time data can be valuable for healthcare providers to effectively and quickly adjust treatment plans.^12^ Additionally, mobile applications aimed at assisting patients with IBD have the potential to enhance care in various ways, including a better understanding of the disease, improved medication adherence, accessible support networks, and early interventions by healthcare providers in case of issues.^20^ They also serve as a promising telehealth tool to facilitate self-management in a new healthcare model, where patients have closer interactions with their medical team and are involved in their decision-making process, increasing opportunities for patient-centered care.^33^

 The results of a study by Con et al,^19^ which examined the content and tools available in IBD applications to identify functions that may assist in patient self-management, indicated a continuous and accurate increase in evaluating applications designed to change behavior for promoting health and disease management. This reflects the growing trend of these applications in the self-management of IBD and the importance of evaluating them.

 Results from a meta-analysis showed that e-health interventions improved self-management in patients with IBD by enhancing their quality of life, reducing psychological distress, and increasing medication adherence. These interventions also significantly reduced the number of clinic or hospital visits for patients with IBD.^34^

 Based on the results of the present study, the least application of IBD management applications was related to the early diagnosis of this disease. The study by Kelso et al^20^ indicated that less than 10% of mHealth apps focus on providing diagnostic tools.

 The lack of diagnostic features may stem from several factors. The complexity of accurately diagnosing IBD through an app without clinical intervention poses a challenge. IBD symptoms often overlap with other gastrointestinal disorders, making differential diagnosis difficult without professional assessment. Additionally, regulatory concerns and privacy issues regarding the management of sensitive health data may deter developers from incorporating robust diagnostic tools.^35,36^ Despite these challenges, the importance of early diagnosis cannot be overlooked. Early diagnosis of IBD can prevent complications, reduce the need for extensive surgery, and improve overall disease management. Therefore, there is a clear need for innovation in this area, potentially focusing on developing non-invasive and user-friendly diagnostic tools that can be integrated into mHealth applications. Such advancements could bridge the current gap and provide comprehensive care for patients with IBD, from prevention and diagnosis to treatment and self-management.

 Based on the results of the current study, one of the features of IBD management applications is educating patients to manage their condition, which includes teaching a healthy lifestyle such as nutrition, exercise, physical activity, maintaining calmness and mental health, coping strategies for fatigue, and disease knowledge. Previous studies have shown that good knowledge about IBD plays a vital role in the self-management of this disease and positively impacts treatment acceptance, optimization, adherence, and social well-being.^19,37,38^ Norouzkhani and colleagues,^39^ in their study aimed at examining the informational and supportive needs and identifying gaps related to these needs in patients with IBD, concluded that the most important supportive needs of these patients are educational needs, including nutrition, medication side effects, surgical complications, and long-term disease outcomes. These needs align with the features utilized in the applications examined in the present study. In another study by Khalil et al,^40^ aimed at identifying unmet educational needs of patients with IBD, it was found that the most important educational need, in addition to the aspects mentioned earlier, is guidance on insurance coverage and education for family and friends, as many patients experience a lack of empathy and understanding from those around them. The management of patients with IBD should not only focus on medical aspects but also provide education on psychological and social dimensions, enhancing the quality of life for patients and their ability to participate entirely in their self-management.^41^ It is recommended that future educational content in applications be designed with these considerations in mind.

 Another feature of the IBD management application in this study is patient monitoring through a direct connection to the IBD Registry, linking to HIS for effective use of health data by physicians, and EMR for reviewing clinical deviations and disease flare-ups. Maintaining communication between patients and physicians during disease exacerbations and direct monitoring by nutrition specialists over patients’ diets are also relevant features of the applications in this study.

 The direct connection of IBD management applications to the IBD Registry provides the capability for real-time tracking of patient data and trends in a broader population, which can aid in understanding treatment effectiveness and conducting research for better management strategies. Additionally, by connecting applications to HIS, physicians can access patient health data more effectively, leading to more personalized and timely care.^18,42,43^

 Maintaining communication between patients and healthcare providers during disease flare-ups is critical, ensuring that patients receive the necessary support and guidance for effective symptom management. Furthermore, the involvement of nutrition specialists in monitoring and advising patients regarding their diet can lead to better disease control and improved quality of life for patients with IBD.^16,44^

 Collectively, these features contribute to a more comprehensive and preventive approach to managing IBD and emphasize the importance of continuous monitoring, personalized care, and the integration of various healthcare specialists in the treatment process for these patients. This model has the potential to improve patient outcomes and satisfaction.

 Visual reports generated as charts from data related to disease symptoms, medication adherence, and diet through mobile applications, integrated with EHRs in patients with IBD, have great potential to enhance the monitoring and management of this chronic condition.^45^ Patients with IBD can gain better control over their condition by tracking their symptoms and sharing this information in real time with their healthcare team. This can lead to more personalized treatment plans and quicker interventions when necessary, ultimately improving the quality of care for patients with IBD.^16,46,47^

 Another feature of IBD management applications is counseling and treatment, which includes collecting health data from patients, tracking patient symptoms and provider feedback, offering evidence-based care, electronic cognitive behavioral therapy, and decision support systems to create alerts.

 Counseling and treatment through mobile applications enable patients to receive medical consultations and treatment plans directly through their smartphones, making this advice accessible to patients in remote locations. Continuous symptom tracking aids in the early detection of flare-ups and facilitates timely interventions that can prevent complications and hospitalizations.^15,20,45^ Utilizing evidence-based guidelines in disease management applications ensures that the care provided is current and effective through research. Additionally, cognitive-behavioral strategies can help patients cope with the psychological impacts of IBD. Applications equipped with alert systems can notify patients and providers about potential issues, such as medication adherence or symptom exacerbation, allowing for prompt actions.^16,48^

 These features collectively enhance the quality of care for patients with IBD by facilitating better self-management, improving communication between patients and providers, and ensuring that treatment decisions are evidence-based. Studies have indicated that such comprehensive apps can significantly improve user-reported disease management levels, and top-rated apps often include a variety of these features.^49^ Integrating these features into IBD management apps can improve health outcomes and a better quality of life for patients with this chronic disease.

 The ability to assess disease severity is another feature of the IBD management application. Home tests measuring fecal calprotectin using smartphones are utilized to determine the level of inflammation and disease flare-ups. Artificial intelligence, employing advanced algorithms and machine learning, can analyze patient data, thereby increasing the accuracy of IBD diagnosis, grading disease severity, and determining its extent.^50-52^ In line with patient-centered care, empowered patients can use new home calprotectin tests that are sufficiently accurate and useful to rule out disease activity at low levels of fecal calprotectin. These new tools can be one of the first steps toward comprehensive health in IBD. In the near future, machine learning may enable the integration of large datasets into personalized algorithms.^37,53^

 Other results from the present study showed that the technologies used in the reviewed applications include connections to EMR, EHR, and hospital HIS, the use of artificial intelligence techniques such as machine learning and data mining, automated messaging, cloud computing, data encryption, barcode scanning, and the connection of smartwatch applications to mobile devices. Smartphone applications are suitable tools that create a platform for remote monitoring of health parameters. More integrated platforms that directly input health data into the EHR can be accompanied by expert systems and decision support tools for semi-automatic review and interpretation of a wide array of submitted data, enabling continuous monitoring and management of patient health beyond outpatient or inpatient visits.^18,45,54^

 Applications of artificial intelligence in IBD cover recent advancements in interpreting and scoring endoscopic images, new capabilities for analyzing cross-sectional images, natural language processing for automatic understanding of clinical text, and improvements in clinical decision support tools.^53^

 Through mobile computing based on cloud computing, large and complex calculations can be performed on powerful cloud servers rather than on mobile devices with limited resources. This maintains the application’s performance, which includes fulfilling requested services, data storage, and energy efficiency while also improving processor capabilities and significantly reducing the energy required by mobile devices.^55,56^

 The use of barcode scanner technology in IBD disease management applications can simplify the process of recording medications or dietary supplements by scanning barcodes, making it easier for patients to track their consumption levels.^36,57^ Additionally, using smartwatch connectivity technology can provide real-time monitoring of health parameters, such as heart rate, which can be beneficial for managing IBD.^36^ Other results from the current study indicated that IBD disease management applications face limitations, including complex implementation across different smartphone types, inconsistencies in user experience due to differences in screen shapes and operating systems, security risks when printing due to the lack of data encryption in QR codes, functionality limited to Microsoft Windows environments and Android phones, and the inability to use the application without basic computer skills and internet access.

 The results of the study by Cucciniello et al,^58^ which aimed to conduct a systematic review of randomized studies on smartphone apps designed for managing high-prevalence diseases, highlight the lack of official development standards in the design process of chronic disease management applications. This study emphasizes policymakers’ and developers’ need to address security issues and ensure continuous software updates. It suggests that users and healthcare professionals should participate in the development process of applications to align their features with the actual needs and preferences of end users.

 Overall, while applications have the potential to assist in managing IBD, improvements in design, security, and accessibility are needed to make them more inclusive and safer for all users. Therefore, it is recommended that developers of these applications consider multi-platform development frameworks, enhance security features, and simplify user interfaces to overcome these limitations.

 The current study faced limitations, including the review of only three databases, which may not have included all relevant studies. Access to the full text of some articles was unavailable, and only English-language articles were considered in the inclusion and exclusion criteria. Nevertheless, the researchers aimed to select studies of sufficient quality by applying strict inclusion and exclusion criteria.

## Conclusion

 Numerous mobile applications have been designed to manage IBD. These applications vary in features and technologies used, with few employing artificial intelligence and cloud computing for self-management of the disease. The use of a single platform for application design and lack of internet access may limit the enthusiasm for using them. Additionally, using QR codes and the lack of data encryption can pose security risks. Existing IBD applications play a limited role in diagnosing recurrences and severity of the disease. Given the importance of early diagnosis and its impact on treatment choices, the current applications may not be well-received by physicians. Despite these limitations, mobile applications have the potential to transform patients into dynamic and empowered individuals in their treatment and disease management through effective education, counseling, and monitoring. It should also be considered that using artificial intelligence and decision-support systems in future designs may enhance the quality and reliability of these applications for disease management, paving the way for personalized medicine.
